# Personal

**Published:** 1890-10

**Authors:** 


### Personal.

DR. WM. C. KRAUSS, of this city, and Miss Krieger, of Salamanca, were united in marriage at the residence of the bride's parents, September 4, 1890.

---

DR. DAGENAIS has removed from his old residence, 348 E. Eagle street, into handsome new quarters, which are located at 473 Virginia street.

Dr. Stockton has returned from Europe, and is occupying offices with Dr. Jones, corner Virginia and Franklin streets, pending the completion of his beautiful new home, 434 Franklin street.

---

Dr. H. Y. Grant has taken offices in the Bachelor building, No. 30 West Tupper street.

